# Fragment Autoantigens Stimulated T‐Cell‐Immunotherapy (FAST) as a Fast Autologous Cancer Vaccine

**DOI:** 10.1002/advs.202502937

**Published:** 2025-03-26

**Authors:** Yuan Li, Huiqin Chen, Qiaofeng Shen, Yingshuang Liu, Pingping Li, Yuqi Ma, Yugang Wang, Shengkai Li, Xueqing Yan, Liyu Liu, Jianwei Shuai, Min Wu, Qi Ouyang, Feng‐Ming (Spring) Kong, Gen Yang

**Affiliations:** ^1^ State Key Laboratory of Nuclear Physics and Technology School of Physics Peking University Beijing 100871 China; ^2^ Wenzhou Institute University of Chinese Academy of Sciences Wenzhou 352001 China; ^3^ Department of Physics Princeton University Princeton NJ 08544 USA; ^4^ Center for Quantitative Biology Peking University Beijing 100871 China; ^5^ Department of Clinical Oncology University of Hong Kong Hong Kong 999077 China

**Keywords:** immunotherapy, radiotherapy, whole tumor cell vaccines

## Abstract

Tumor cells often down‐regulate antigen presentation and mount an immunosuppressive microenvironment, hindering successful cancer immunotherapy and vaccine development. Additionally, due to genomic instability, tumor cells are usually heterogeneous and constantly evolving. Therefore, vaccines need broad antigen coverage and rapid preparation. Here, a personalized whole tumor cell vaccine (TCV), termed fragment autoantigens stimulated T‐cell‐immunotherapy (FAST) is developed. In 7 h, tumor cells are treated with irradiation and cryoablation. Personalized fragmented antigens (FAs) from these treated cells are used as TCVs. In breast, colon, and melanoma mouse models, FAST achieved significant tumor regression, less metastasis, and longer survival. Notably, FAST outperforms other advanced TCVs, especially in curbing metastasis. Mechanistically, FAs activate efficient, broad‐spectrum antigen presentation due to upregulation of immunogenic cell death, MHC‐I, and damage‐associated molecular patterns. Concurrently, FAST also enhances anti‐tumor immunity by reshaping immune microenvironments. Analysis of clinical data shows FAST‐associated proteins have prognostic and therapeutic value in patients with liver, stomach, rectal cancers, and melanoma. These results suggest FAST has high anti‐tumor efficacy and potential as a personalized TCV platform. The relevant clinical trial NCT06756295 is under initiation with approval of ethics.

## Graphical abstract

A personalized whole tumor cell vaccine (FAST) is developed to address immunosuppressive tumor microenvironments and genomic instability‐driven heterogeneity. Irradiation and cryoablation‐treated tumor cells generate fragmented antigens (FAs) that activate broad‐spectrum antigen presentation via upregulated immunogenic cell death, MHC‐I, and damage‐associated molecular patterns. FAST demonstrates superior tumor regression, metastasis inhibition, and survival benefits in murine models compared to advanced vaccines, while reshaping immune microenvironments. Clinical trial NCT06756295 is initiated.

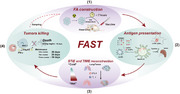

## 1 Introduction

Tumor vaccines have long been considered promising tools for cancer immunotherapy by harnessing host immunity against tumor cells and being able to deliver tumor‐specific antigens to antigen‐presenting cells, thereby stimulating the immune system and triggering an effective immune response against the tumor.^[^ ^1^, ^2^, ^3^ ^]^ However, the high heterogeneity, complexity, diversity, and mutagenicity of tumor antigens result in a very limited number of shared antigens between different cancers, which is insufficient for universal cancer vaccine preparation.^[^ ^4^ ^]^ In contrast, personalized vaccines based on neoantigens require the prior identification and preparation of neoantigens, a complex and time‐consuming process in which patients may lead to disease progression. Whole tumor cell vaccines (TCVs) composed of whole tumor antigens have received significant attention as reliable and effective vaccines for tumor prevention and treatment.^[^ ^3^ ^]^ In recent years, significant progress has been made in the pre‐clinical development of whole tumor cell vaccines,^[^ ^5^, ^6^, ^7^ ^]^ and several vaccines, including the melanoma whole tumor cell vaccine,^[^ ^8^, ^9^ ^]^ have entered or are undergoing clinical trials. However, the vast majority of these vaccines have failed to translate into clinical applications, and no whole tumor cell vaccine has yet been approved by the FDA. The main reason for this failure is the low recognition and limited abundance of tumor antigens, coupled with insufficient exposure of internal tumor antigens. These factors result in the tumor cells’ poor immunogenicity and the ineffective activation of the immune system to clear the tumor.^[^ ^10^ ^]^

At the same time, immunosuppressive cells, such as regulatory T cells, myeloid‐derived suppressor cells (MDSC) and cancer‐associated fibroblasts in the tumor immune microenvironment (TIME) release a large number of immunosuppressive signals into the microenvironment, including programmed death‐ligand 1 (PD‐L1), transforming growth factor‐beta (TGF‐β), and vascular endothelial growth factor, and directly or indirectly act on the effector T cells, creating an environment that inhibits the TIME, reduces the efficacy of the treatment, and protects the tumor from immune attack.^[^ ^11^, ^12^ ^]^ In addition, prolonged exposure to tumor antigens during cancer progression leads to T‐cell depletion, and immunosuppressive cells generated during treatment can further weaken the anti‐tumor effects of adaptive immunity.^[^ ^13^ ^]^ This leads to failure of tumor immune rejection, especially in metastatic tumors that rely on immune‐mediated destruction.

Immune checkpoint blockade has achieved considerable success in tumor therapy. However, majority patients show only minimal or no response to these treatments.^[^ ^14^ ^]^ Studies have shown that a suppressive TIME, characterized by low levels of tumor‐infiltrating lymphocytes (TILs) and suppressor immune cells, is a major barrier to effective immunotherapy.^[^ ^15^, ^16^ ^]^ Chow et al. found that T‐cell “depletion” in the TIME, often considered one of the main mechanisms by which the critical role of immune checkpoint inhibitors is diminished, leads to resistance to tumor immunotherapy.^[^ ^17^ ^]^

Studies have shown that radiation therapy, a routine treatment technique for cancer patients, can be used not only to inactivate tumor tissue and reduce its oncogenic potential, but also to play an important role in modulating the tumor immune microenvironment.^[^ ^18^, ^19^ ^]^ Radiation induces tumor cells to produce immunogenic neoantigens,^[^ ^20^ ^]^ simultaneous release of large amounts of double‐stranded DNA (dsDNA),^[^ ^21^ ^]^ damage‐associated molecular patterns (DAMPs), and various cytokines that trigger tumor cells to undergo immunogenic cell death (ICD) and alter cell surface molecules,^[^ ^22^ ^]^ thereby inducing an effective anti‐tumor immune response. Additionally, these processes enhance the uptake of tumor antigens by dendritic cells (DCs),^[^ ^23^ ^]^ promote antigen presentation, and increase T cell activation levels.^[^ ^24^, ^25^ ^]^ However, the effect of radiotherapy on the tumor microenvironment is extremely complex, even exerting opposing effects on the host immune system.^[^ ^26^ ^]^ The effect whereby radiotherapy at one site may lead to regression of metastatic cancer at distant sites that are not irradiated was described and called the abscopal effect. The abscopal effect has been connected to mechanisms involving the immune system. However, the effect is rare because at the time of treatment, established immune‐tolerance mechanisms may hamper the development of sufficiently robust abscopal responses.^[^ ^10^ ^]^

Briefly, as a matter of fact, reduced or lost antigen presentation is a common and necessary mechanism by which tumor cells evade immune recognition and destruction, including genomic deletion of the MHC‐I gene, transcriptional repression of genes involved in antigen presentation, dysregulated processing of tumor antigens, and defects in the translocation of antigens to the cell surface and presentation.^[^ ^27^, ^28^, ^29^ ^]^ Meanwhile, suppressive TIME is also a major barrier to effective immunotherapy.^[^ ^15^, ^16^ ^]^ To address these problems, we have developed an effective anti‐tumor vaccine named FAST, which has been demonstrated in several preclinical mouse tumor models and with clinical data. The FAST process involves the following steps: tumor cells undergo a combination of irradiation and cryoablation cycles within a 7‐h timeframe. Personalized fragmented antigens (FAs) are derived from these tumor cells and subsequently administered to host as TCVs. Due to the activation of the ICD process and its derivation from the whole‐cell processing pathway, FAs can activate highly efficient and broad‐spectrum antigen presentation and recognization. The FAs consist of a complex mixture of biologically active components, including tumor antigens, cell membrane fragments, diverse cytokines, DAMPs, and dsDNA, all of which play pivotal roles in eliciting robust immune responses. FAST also significantly remodels both the TIME and the STIE when administered to host. As a result, FAST can effectively suppress tumor growth, reduce metastasis, and enhance overall survival in pre‐clinical animal tumor models. Our findings indicate that FAST exhibits potent anti‐tumor activity and holds considerable potential as a platform for the development of personalized TCVs. A clinical trial NCT06756295 has been initiated, with ethical approval, to evaluate its therapeutic efficacy and safety in patients.

## 2 Results

### 2.1 FAST Significantly Inhibits Tumor Progression, Decreases Metastasis, and Extends Survival

**Figure 1:**
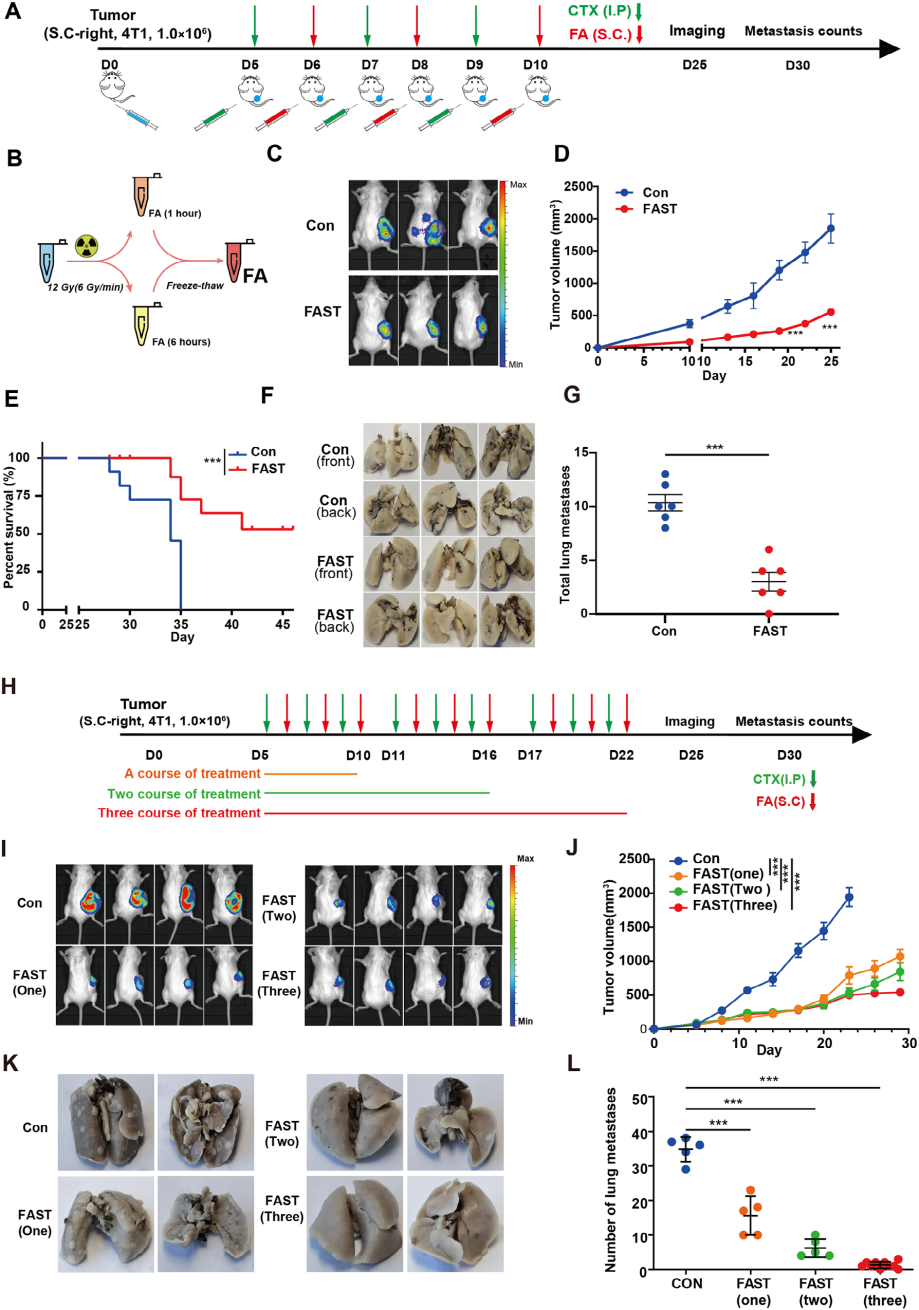
FAST inhibits local tumor progression, distant metastasis and improves overall survival in a mouse breast tumor model. This figure shows the FAST effect on 4T1 mouse breast tumor model. The experimental procedure schematic is shown in (A): D0 inoculating the tumor on the right flank, intraperitoneal injection of adjuvant Cyclophosphamide (CTX) at D5, D7, and D9. Fragment autoantigens are inoculated on the left of the mice at D6, D8, and D10. This cycle is repeated three times for one treatment course. Tumor imaging is done on day 25, and the lung metastasis status of mice is recorded on day 30. (B) FA preparation process. (C) Imaging of the model for distant tumors. The therapeutic effect of FAST on 4T1 tumors (*n* = 6 mice) are shown in tumor growth curves (D) and Survival curve (*n* = 10 mice) (E). Gross appearance of lungs with metastastic tumors harvested (G) and number of total lung surface metastases (G), showing reduction of overall tumor volume and number of distant metastasis. (H) shows multiple courses of FAST treatments: one course (orange), two courses (green), and three courses (red). The effects of multiple course treatment are shown in (I) In vivo imaging of the animal. (J) Tumor growth curves for different FAST treatment courses. (K) Images of lung metastasis in and (L) Quantification of lung metastasis in the multiple treatment regimen mouse model, showing significant differences from 1 to 3 courses of treatment. Statistical significance was analyzed by Student's *t*‐test, Log Rank Test, and two‐way ANOVA (Tukey). *p*(***) < 0.001, *p*(**) < 0.01, *p*(*) < 0.05 versus CON, etc.

We report the development of a rapid and efficient therapeutic approach, FAST. This method uses irradiation to transition tumors from a low‐immunogenic state to a high‐immunogenic state, while preserving all tumor antigens (**Figure** **1**B). This method also employs freeze‐thaw treatment to eliminate tumorigenicity and is combined with adjuvant therapy to treat metastatic tumors. To validate the inhibitory efficacy of FAST on established tumors, we constructed a distant tumor model of 4T1 breast cancer. When the primary tumor reached ≈150 mm^3^, FAST treatment was administered on the contralateral side, where no tumor was present (Figure 1A). We found that either Adjuvant or FAST effectively suppressed the growth of distant tumors (Figure 1D; Figure S1A, Supporting Information), and more than 50% of FAST‐treated mice still survived at 45 days post‐treatment, while all mice of the control group died at 35 days post‐treatment (Figure 1E; Figure S1C, Supporting Information). Similar results were obtained from melanoma B16‐F10 tumor (Figure S1G–I, Supporting Information) and colon cancer CT‐26 models (Figure S1J–L, Supporting Information). In B16F10 model, mice in the control, adjuvant, and FA groups all died around day 35, while mice treated with FAST survived for more than 50% (Figure S1G–I, Supporting Information). All mice in the control group died at 40 days after treatment, while the mice in the FAST group remained a 100% survival rate until day 65 post‐treatment (Figure S1L, Supporting Information) in colon cancer CT‐26 models. These results indicate that FAST strongly inhibits tumor growth and prolongs survival.

The primary tumor continuously releases tumor cells, which, as individual circulating tumor cells (CTCs) or CTC clusters, migrate through the bloodstream or lymphatic vessels and eventually colonize tissues to form metastatic neoplasms. Compared to Adjuvant, although FA only exhibited less pronounced inhibition of distant tumor growth, it significantly reduced tumor lung metastasis (Figure S1A–D, Supporting Information). However, the adjuvant treatment effectively inhibited distant tumor growth but simultaneously increased the risk of lung metastasis. Furthermore, the FAST treatment exhibits substantially pronounced effects on suppressing metastasis (Figure 1G; Figure S1A,B,D, Supporting Information). Van Putten et al have reported that cyclophosphamide increased the formation of pulmonary metastasis by up to 1000‐fold in a mouse model.^[^ ^30^, ^31^ ^]^ However, this drug did not have a direct effect on the metastatic capacity of cancer cells. Instead, it enhanced the adhesive properties of the lung vasculature for CTCs through a metalloproteinase‐2‐dependent mechanism that resulted in remodeling of the vasculature basal membrane.

To investigate whether FAST can induce long‐term anti‐tumor effects, we designed various immunotherapy regimens. We found that 1–3 courses of FAST all effectively inhibited distant tumor growth and significantly reduced tumor lung metastasis compared to the control (Figure 1H–L). Notably, mice subjected to 3 times of FAST (FAST‐3) exhibited the best effect on distant tumor control and lung metastasis inhibition. One month after treatment, FAST‐3 displayed 100% tumor growth control (Figure 1I,J), resulting in almost complete suppression of lung metastasis (Figure 1K,L). Combined with the above results, we found that the FAST‐1 (one‐course) group (orange) demonstrates an increase in tumor growth rate at the day 20 post‐inoculation. Similarly, the FAST‐2 (two‐course) group (green) exhibits a moderate increase in tumor growth rate around day 25 post‐inoculation. In contrast, the FAST‐3 (three‐course) group (red) shows a stabilization in tumor growth around day 30 post‐inoculation (Figure 1J, Graph Abstract 4). Further analysis revealed that the exceptional anti‐tumor effects of FAST‐3 resulted from the induction of central memory T cells (T_cm_) and effector memory T cells (T_em_) (Figure S1E, Supporting Information).

Immune checkpoint inhibitors (ICIs) are one of the mainstays of cancer treatment in recent years. Compared to monotherapy with immune therapy drugs, the mRNA‐4157 vaccine combined with ICI reduced the risk of death or recurrence by 44% in recipients.^[^ ^32^ ^]^ To determine whether the combination of FAST and ICI could yield a higher anti‐tumor effect, we treated tumor‐bearing mice with FA in combination with PD‐L1 or cytotoxic T‐lymphocyte‐associated antigen 4 (CTLA‐4) inhibitors, and found that combination with ICI (α‐PD‐L1 or α‐CTLA‐4) did not significantly enhance the inhibitory effect of FA on distant tumors (Figure S2A–C, Supporting Information). At the end of observation, only the combination with α‐PD‐L1 showed a slight improvement in distant tumor suppression (Figure S2C,E, Supporting Information). Additionally, we found that FA combined with anti‐PD‐L1 did not further attenuate the risk of lung metastasis, whereas combined with anti‐CTLA‐4 did reduce the risk of lung metastasis (Figure S2E–G, Supporting Information).

These results demonstrate that FAST exerts significant anti‐tumor immune effects. After multiple rounds of treatment, it generates immune memory and enhances metastatic inhibition. When combined with immune checkpoint blockade therapy (anti‐CTLA‐4), it may further strengthen the suppression of cancer metastasis.

### 2.2 FAST Reshapes the Immune Microenvironment in the Tumor and the Lung

The significance of TIME and STIE in regulating cancer progression and affecting therapeutic outcomes is now widely accepted.^[^ ^33^ ^]^ The high heterogeneity of the TIME is a major challenge in treating cancer. The presence of TILs in tumors is associated with improved clinical prognosis. Nevertheless, whether the tumor is ultimately controlled or progresses depends on the type,^[^ ^34^ ^]^ function, and localization of various TILs in the TIME. As a form of immunotherapy, we investigated whether FAST could potentially reshape the TIME and STIE. As the lung is a common site for metastasis and is often targeted in advanced solid tumors, we further investigated the potential of “re‐educating” the immune microenvironment both in the primary tumor and in the lung metastasis.

**Figure 2:**
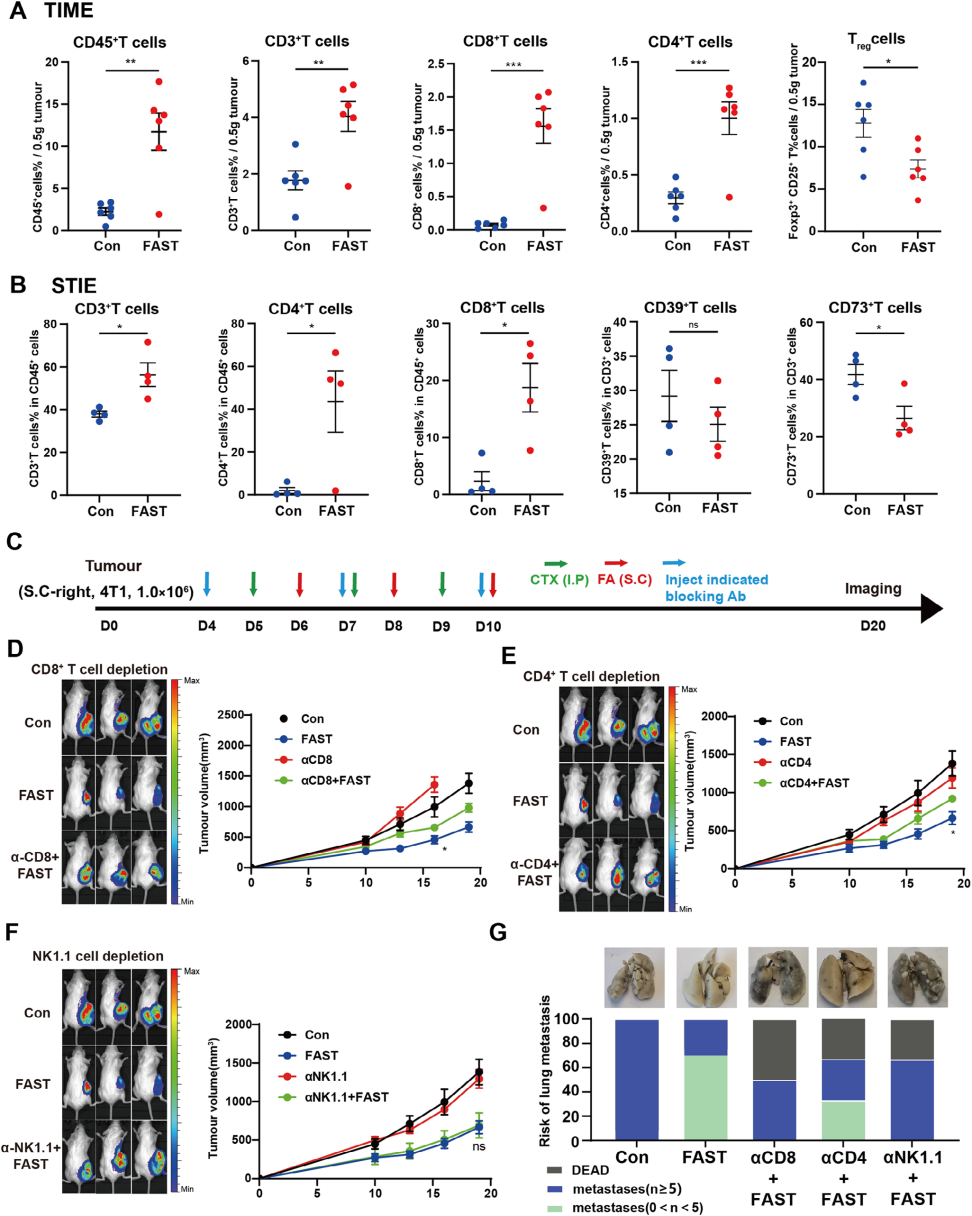
FAST induces antitumor responses through activating of CD4+ and CD8+ T cells and deceasing of suppressive T cells. (A) shows that on day 30 post‐tumor implantation, after FAST treatment, the tumor‐infiltrating T cell populations, including CD45^+^, CD3^+^, CD8^+^, and CD4^+^ T cells, were significantly increased, while Foxp3^+^CD25^+^ T_reg_ cells were significantly decreased. (B) On day 30 post‐tumor implantation, similar results were observed in the lungs after FAST treatment, with tumor‐infiltrating T cell populations, including CD3^+^, CD4^+^, and CD8^+^ T cells, significantly increased, while CD73^+^ immunosuppressive T cells were significantly decreased. (C–G) shows the contribution of CD4^+^ T cells, CD8^+^ T cells, and NK cells to the FAST‐induced anti‐tumor efficacy. D0 inoculating the tumor on the right flank. At D0, D7, and D10, mice were immunized with or without anti‐CD8, anti‐CD4, and anti‐NK cells respectively. Also, adjuvant CTX is intraperitoneal injected at D5, D7, and D9. Fragment autoantigens are inoculated on the left of the mice at D6, D8, and D10 (*n* = 5). Presented are the fluorescence intensity during the live imaging process (E,F), as well as the tumor growth curves and the situation of lung metastasis (G).

To elucidate the anti‐tumor mechanism of FAST, we detected TIME in tumor tissues by flow cytometry. We focus on detecting its immune infiltration, specifically the infiltration of CD45+ lymphocytes, and thus CD45^+^ is used as one of the indicators in the TIME analysis. We found that FAST significantly increased the percentages of leukocytes and lymphocytes in the tumor (**Figure** **2**). Further analysis revealed an increase in tumor‐infiltrating CD8^+^ and CD4^+^ T cells in the FAST group, along with a reduction in the proportion of regulatory T (T_reg_) cells (Figure 2A). These findings suggested that FAST activates specific immune responses while improving the TIME, transforming “cold” tumors into “hot” tumors, thereby enhancing the anti‐tumor efficacy. FAST also increased the percentages of Ki67^+^ T cells and IFN‐γ^+^ T cells (Figure S3A,B, Supporting Information), suggesting that FAST promotes T cell proliferation and anti‐tumor ability.

To investigate the mechanism of FAST inhibiting lung metastasis, we analyzed lung tissues of mice (STIE) and found that CD3^+^/CD8^+^/CD4^+^ T cells were increased in the lungs after FAST treatment (Figure 2B). Our primary focus is on the changes in the immune ecology before tumor metastasis in the STIE, with CD39/CD73 serving as key molecular markers of the STIE's inhibitory status. Whereas a significant decrease in the suppressive phenotype of CD73^+^ was observed, and a decreasing trend of CD39^+^ was also present (Figure 2B). This signifies that FAST treatment reduced immune suppression in the lung and concurrently upregulated cytotoxic T lymphocytes, suggesting the anti‐tumor immune response within the lung was enhanced after FAST treatment. Notably, CTX alone also significantly inhibited immunosuppressive lymphocytes CD73^+^ but failed to enhance lung cytotoxic T lymphocytes (Figure S3C–E, Supporting Information), indicating that FAST was more conducive to exerting a stronger anti‐tumor effect on the lung microenvironment.

To elucidate the functions of CD4^+^ T cells, CD8^+^ T cells and NK cells within the context of the anti‐tumor response elicited by FAST, we utilized specific depletion antibodies to monitor the anti‐tumor efficacy of FAST following the depletion of the respective immune cells (Figure 2C–G). Compared to the control group, the sole depletion of either CD4^+^ T or CD8^+^ T cells did not modify tumor growth dynamics (Figure 2C–G). However, compared to the FAST‐treated groups, the depletion of CD4^+^ T or CD8^+^ T cells partially diminished the anti‐tumor effect of FAST (Figure 2D,E), suggesting that both CD4^+^ T and CD8^+^ T cells play critical roles in suppressing tumor progression in the FAST treatment group. Notably, the antagonism of NK cells exerted no substantial influence on tumor growth in the FAST‐treated group (Figure 2F). However, the depletion of NK cells, CD4^+^ T cells, or CD8^+^ T cells all led to an augmented incidence of lung metastasis (Figure 2G), signifying that these cells assume significant roles in control of tumor lung metastasis.

### 2.3 DAMPs Released in the FA Bolster the Immune Response by Facilitating Dendritic Cell Maturation and Activating T Cells

Radiation induces immunogenic cell death in tumor cells, leading to the release or exposure of DAMPs, such as calreticulin (CRT), dsDNA, ATP, and HMGB1. These DAMPs drive, activate, attract, and stimulate antigen‐presenting cells especially DCs through various pattern‐recognition receptors, ultimately cross‐presenting tumor antigens to NK cells and T cells, and activating specific immune responses.^[^ ^22^, ^23^ ^]^ Studies have shown that the increased level of HMGB1 in blood after radiotherapy and chemotherapy is associated with a good prognosis in head and neck squamous cell carcinoma, and HMGB1 can be used to predict the outcome of malignant tumors.^[^ ^35^ ^]^

**Figure 3:**
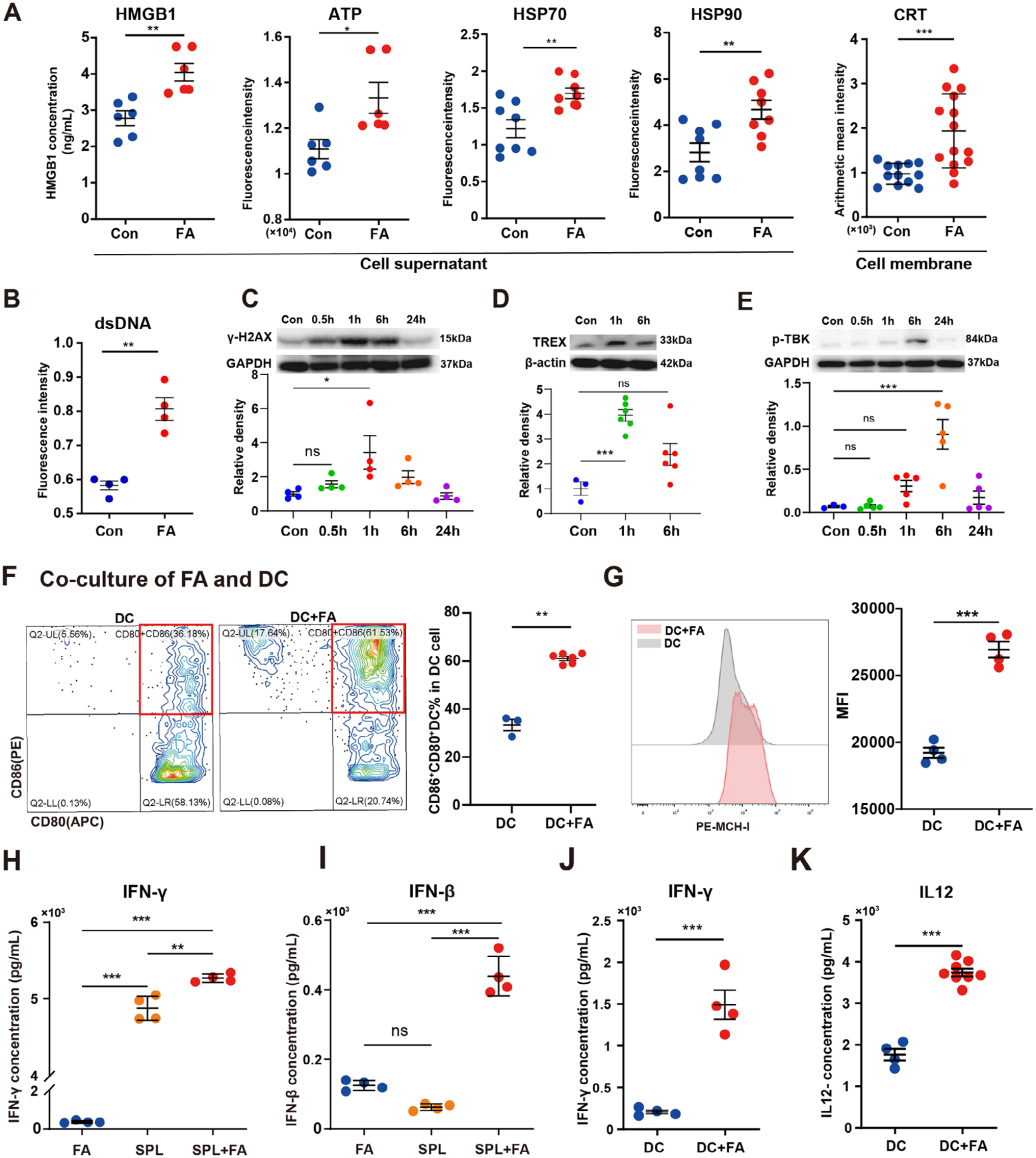
The pronounced secretion of DAMPs within the context of FA led to an enhanced maturation of DCs and augmented cytokine secretion. (A) demonstrates that the levels of ICD markers in FA, namely HMGB1, ATP, HSP70, HSP90, and CRT, exhibit a significant elevation at 6 h subsequent to irradiation. (B) reveals that the cytoplasmic dsDNA content undergoes a marked increase 6 h post‐radiation. (C) illustrates the nuclear DNA damages as indicated by γH2AX at various time intervals subsequent to radiation exposure. (D) illustrates that the expression levels of TREX manifest a significant elevation at 1 and 6 h following irradiation. (E) displays the substantially up‐regulated expression profiles of p‐TBK in the downstream of the cGAS‐STING pathway at 1 and 6 h post‐irradiation. (F) depicts an elevated maturation degree of DCs subsequent to co‐culture with FA. (G) demonstrates that the surface abundance of MHC‐I, denoted as MHC‐I fluorescence intensity (MFI), on DCs is augmented after co‐culture with FA. (H,I) reveal a remarkable augmentation in the secretions of IFN‐γ and IFN‐β upon co‐culturing splenic lymphocytes (SPL) with FA for 24 h. (J,K) exhibit a significant elevation in the secretion levels of IFN‐γ and IL‐12 following the 24‐h co‐culture of DCs with FA. Statistical significance was analyzed by Students' *t*‐test or two‐way ANOVA. *p*(***) < 0.001, *p*(**) < 0.01, *p*(*) < 0.05 versus Con etc.

To uncover the reasons behind the high immunogenicity of FA, we examined the levels of DAMPs in suspensions from irradiated cells before cryoablation, and found that HMGB1 secretion was significantly higher in FA compared to the control (**Figure** **3**A). Meanwhile, ATP was also significantly increased in the FA group (Figure 3A). The changes in the levels of HSP70 and HSP90 in irradiated cell suspensions were consistent with those of HMGB1 and ATP (Figure S8A,B, Supporting Information), especially at 6 h after irradiation (Figure 3A). CRT acts as an “eat me” signal to stimulate antigen‐presenting cells initiate immune responses.^[^ ^36^ ^]^ More CRT exposure in FA (Figure 3A; Figure S8E, Supporting Information) contributes to the activation of T cell‐mediated anti‐tumor effects. At 6 h of radiation, DNA damage was significantly elevated, and the expression profile of p‐TBK downstream of the cGAS‐STING pathway was significantly upregulated (Figure 3B–E). Based on these results, we suggest that radiation enhances the exposure of CRT and the secretion of HMGB1, dsDNA, ATP, HSP70, and HSP90 by tumor cells, thereby increasing the immunogenicity of FA.

To elucidate whether FA can activate DCs and subsequently enhances the anti‐tumor response in vitro, we examined the immunogenicity of FA. FA or tumor cells lysates were respectively co‐cultured with murine bone marrow‐derived dendritic cells (BMDCs) to detect induced mature BMDC, and lipopolysaccharide (LPS) was used as a positive control. Compared to tumor cells lysates, FA significantly increased the proportion of mature BMDCs, which was comparable to the maturation level of LPS‐induced BMDCs (Figure 3F; Figure S8F, Supporting Information). The levels of IL‐12, IFN‐γ, and IFN‐β secreted by mature DCs were also significantly increased, indicating FA promotes DC maturation and secretes T cell activation factors (Figure 3J,K). Meanwhile, we co‐incubated FA with splenic lymphocytes and found that FA significantly increased the secretion of type I (IFN‐β) and type II (IFN‐γ) interferon from lymphocytes. These results suggested that FA may induce T cells activation and initiated adaptive immune responses by augmenting the secretion of IFN‐β and IFN‐γ (Figure 3H,I). DCs present exogenous antigens on MHC‐I molecules, which is considered a primary mechanism by which DCs initiate tumor‐specific CD8^+^ T cell responses. Further analysis revealed that the expression of MHC‐I‐like molecules on the surface of DC cells increased after FA treatment. The aforementioned results imply that FA has the capacity to augment the antigen presentation of DCs to T cells, trigger the activation of T cells (Figure 3G), and instigate adaptive immune responses through the elevation of DC maturation and the upregulation of the expression of MHC‐I molecules on their surface. In summary, these findings demonstrate that the enhanced secretion of DAMPs within FA serves to potentiate the immune response by facilitating dendritic cell maturation and T cell activation.

### 2.4 FAST Induces Anti‐Tumor Immune Response Through Activating the PI3Kδ and IFNGR1

**Figure 4:**
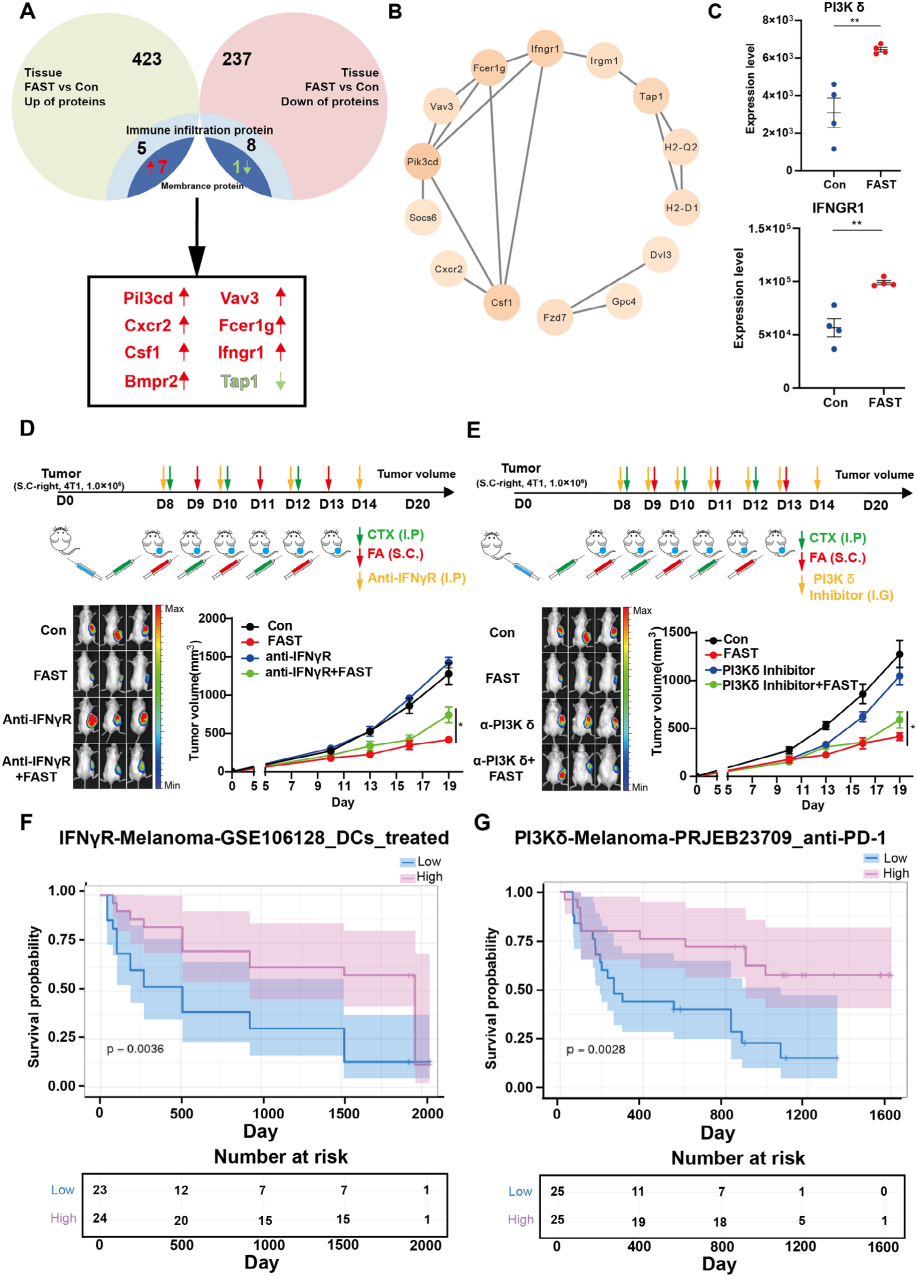
Illustrates the molecular mechanisms underlying the immune responses elicited by the FAST treatment. (A) The analysis of differential membrane proteins between the Con group and the FAST group divulges that there are 12 upregulated and 9 downregulated membrane proteins within the FAST‐treated cohorts. (B) The protein‐protein interaction (PPI) network analysis has identified PI3Kδ as a central gene, which exhibits a correlation with IFNGR, thereby suggesting an interaction between the two proteins. (C) demonstrates the expression level of immune infiltration‐associated membrane proteins in the FAST‐treated group is higher than that in the Con group. (D,E) depicts the diminished capacity of FAST therapy to restrain distant tumors within the IFNγR and PI3Kδ antagonism models. (D) In the IFNγR antagonism model, the fluorescence intensity in the in vivo imaging (left) and the tumor growth curves (right) are exhibited. (E) In the PI3Kδ antagonism model, the fluorescence intensity in the in vivo imaging (left) and the tumor growth curves (right) are displayed. (F) It is demonstrated in the GSE106128 clinical studies that patients with a higher level of IFNγR expression enjoy a better survival prognosis. (G) The PRJEB23709 clinical studies reveal that patients with elevated PI3Kδ expression have a better survival prognosis. Statistical significance was evaluated using the Student's *t*‐test or two‐way ANOVA. *p* (***) < 0.001, *p*(**) < 0.01, *p*(*) < 0.05 versus Con etc.

To unravel the molecular mechanisms that underpin the immune activation against tumors induced by FAST, transcriptomic and proteomic assays were carried out on the tumors from mice subjected to either FAST therapy or the absence thereof. Additionally, the protein expression levels of immune infiltrates within tumors (Con in contrast to FAST) were also analyzed, with membrane proteins serving as the screening benchmarks. The ImmPort Shared Data were utilized for the screening of immune‐related proteins (**Figure** **4**A). By conducting a comparison of the differential gene expression patterns between the tumors under FAST treatment and the control, the overlapping upregulated membrane proteins and immune pathways within the FAST groups were accurately delineated. Twelve shared genes, namely Pik3 cd, Dvl3, Vav3, Fzd7, Gpc4, Cxcr2, Fcer1 g, Socs6, Elmo1, Csf1, Bmpr2, and Ifngr1, were discerned (Figure 4A). In relation to the downregulated membrane proteins in the FAST‐treated tumors, nine co‐expressed genes, specifically H2‐Q2, H2‐D1, Plpp2, Ehd3, Tmem9, Tap1, Irgm1, Slc12a7, and Cnih4, were pinpointed (Figure 4A).

To further ascertain the detailed clustering of the aforementioned proteins, Gene Ontology (GO) and Kyoto Encyclopedia of Genes and Genomes (KEGG) analyses disclosed that these proteins are abundantly represented in adaptive immune processes and associated signaling pathways, such as leukocyte chemotaxis and cytokine‐cytokine receptor interaction (Figure S5A,B, Supporting Information). Through protein‐protein interaction (PPI) network analysis, essential membrane proteins like Pik3 cd, Ifngr1, and Tap1 were singled out (Figure 4B). Moreover, within the set of 12 upregulated proteins, PI3Kδ, a pivotal regulatory factor in both adaptive and innate immunity, was noted to be substantially upregulated in tumors treated with FAST (Figure S4A, Supporting Information). Remarkably, the TCR signaling pathway facilitates T cell survival and cell cycle progression by activating AKT‐dependent transcriptional programs.^[^ ^37^ ^]^ This finding implies that the PI3Kδ – AKT signaling pathway might be implicated in the activation of immune cells triggered by FAST treatment. The IFNγR signaling pathway represents an essential molecule in solid tumor immunotherapy.^[^ ^38^ ^]^ It is markedly upregulated in the FAST‐treated groups (Figure S4A, Supporting Information), suggesting a connection between IFNγR and the anti‐tumor immune effects of FAST. Collectively, the results indicate that PI3Kδ and IFNγR are likely to play critical roles in the anti‐tumor immune process of FAST.

To further validate the critical role of Pik3 cd and Ifngr1 in the anti‐tumor immune effects of FAST therapy, we carried out the inhibition of PI3Kδ in leukocytes and IFNγR in tumor cells. Subsequently, it was found that this intervention led to a substantial attenuation in the ability of FAST therapy to suppress distant tumors (Figure 4D,E). Next, we delved into the impact of PI3Kδ and IFNγR expression on patient prognosis and the response to ICB treatment by leveraging the Tumor Immunotherapy Gene Expression Resource (TIGER) database. Our findings indicated that patients with a higher expression level of PI3Kδ and IFNγR during immunotherapy demonstrated significantly improved survival outcomes (Figure 4F,G). Moreover, we examined the disparities in the expression levels of PI3Kδ and IFNγR between immunotherapy‐responsive patients and non‐responsive patients. It was observed that the expression levels of PI3Kδ and IFNγR in responsive patients were remarkably elevated compared to those in non‐responsive individuals (Figure S6, Supporting Information). Through Hazard Ratio (HR) analysis (HR < 1, *p* < 0.05), we also unearthed that patients with a high expression of PI3Kδ and IFNγR had a more favorable prognosis (Figure S7, Supporting Information). These results collectively imply that PI3Kδ and IFNγR are crucial molecules within the FAST‐induced anti‐tumor immune response.

### 2.5 Potential Antigens Activated in FAST

Radiation has the ability to induce the expression of pre‐existing mutant proteins compared to normal cells. In the cancer cells treated with combined irradiation and cryoablation cycles (the FA construction process), these mutant proteins are potentially neoantigens or associated antigens. They hold the potential to trigger an immune response and expand the immunotherapeutic window for cancers characterized by low mutational loads.^[^ ^39^ ^]^

To pinpoint the potential tumor antigens associated with FA and assess their effectiveness in the tumors of FAST‐treated mice, we carried out comprehensive proteomic and transcriptomic analyses. The aim was to characterize the proteins that were upregulated as a result of the FA construction process.

**Figure 5:**
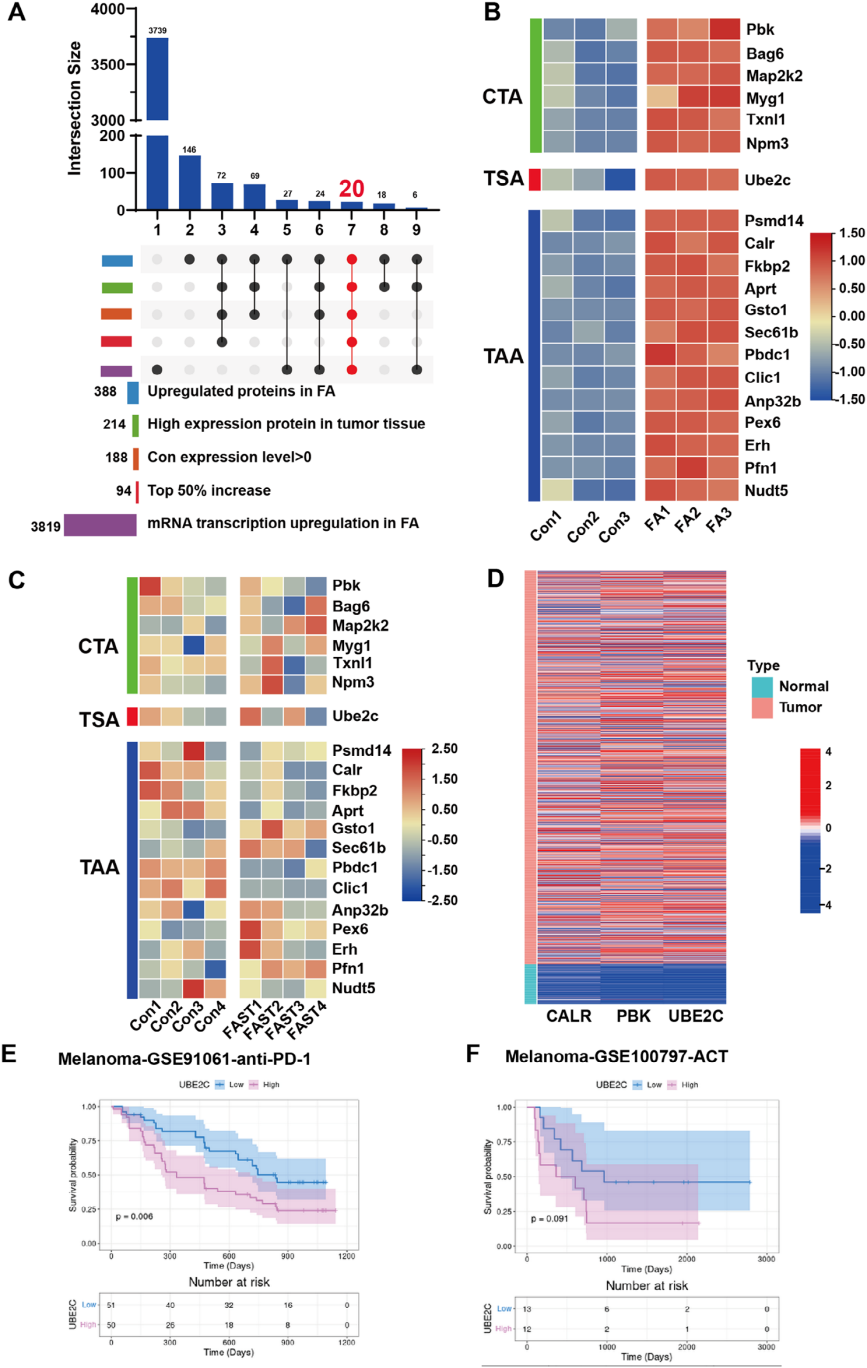
FA‐upregulated tumor antigens may serve as clinical prognostic markers. (A,B) Radiation‐induced antigen prediction screening process in cells. In FA, 388 proteins were upregulated, with 214 showing high expression in tumors. After selecting proteins with values >0 in the control group and in the top 50%, 20 proteins were concurrently upregulated at both the protein and transcript levels (B). (C) After FAST treatment, 20 high‐expression proteins in mouse tumor tissues show a downregulation trend, with statistical tests indicating that there are no significant differences in the expression levels of these 20 proteins between Con and FAST. Tumor cells expressing these proteins may be cleared by the immune system. The upregulated 20 proteins in FAST were analyzed based on TCGA breast cancer (1056 patient sample data) and Differential Expression analysis in normal breast tissue, showing significant upregulation in cancer patients (D). (E,F) High expression of UBE2C is associated with poor prognosis in melanoma patients with ICB.

During this process, these proteins had to meet three crucial criteria:

1. Prior to the FA construction process, their expression levels were higher in cancer cells compared to normal cells.
2. In response to the FA construction process, they were upregulated at both the proteomic and transcriptomic levels.
3. They were among the top 50% in terms of protein expression levels (**Figure** **5**A).

By applying these criteria, we successfully identified 20 proteins that were upregulated by the FA construction process (Figure 5A,B).To classify the antigens as potential cancer‐testis antigens (CTA), tumor‐specific antigens (TSA), or tumor‐associated antigens (TAA), we utilized the GTEx database to examine their expression in normal tissues (Figures S10,S11B, and S12, Supporting Information). Notably, six of these proteins (PBK, BAG6, MAP2K2, MYG1, TXNL1, NPM3) showed high expression levels in testicular tissue but low or even negligible expression in adult tissues, as marked by the red box in the Figure (Figure S10, Supporting Information). This indicates that these six proteins might be potential CTAs. In fact, PBK has been previously reported as a well‐known CTA.

Furthermore, we conducted differential gene expression (DGE) analysis on the TCGA dataset. Four independent algorithms verified the differential expression of these 20 proteins between cancerous and normal tissues, with a significant upregulation in cancer. This analysis also identified three differentially expressed genes: UBE2C, CALR, and PBK (Figure 5D). Among them, UBE2C was identified as a common protein across all four algorithms. Based on the GTEx database analysis, UBE2C was found to be highly expressed in testicular tissue. Paired analysis of patient samples further demonstrated that UBE2C is highly expressed across various cancer types, suggesting that UBE2C could function as a TSA (Figure S11, Supporting Information).

Moreover, we further investigated the expression of these proteins in the tumor tissues of mice after FAST treatment. A general trend of downregulation of these proteins was observed (Figure 5C), suggesting that tumor cells with high antigen expression may have been recognized and, to a certain extent, eradicated by the activated immune system.

To further investigate the clinical relevance of these 20 proteins in patient prognosis, we observed that high expression of these proteins across eight distinct cancer types was associated with poorer prognosis, as evidenced by reduced overall survival (OS) and disease‐free survival (DFS). These findings suggest that these antigens could serve as potential therapeutic targets in these cancers (Figure S9, Supporting Information). By activating the immune system to target and eliminate tumor cells with high expression of these antigens, FAST treatment may have the potential to enhance patient survival prognosis. Further analysis of UBE2C expression revealed that patients with low UBE2C expression exhibited better survival outcomes, particularly in melanoma patients receiving immune checkpoint blockade (ICB) therapy (Figure 5E,F), highlighting UBE2C as a potential risk gene. In summary, UBE2C appears to be a tumor‐specific, high‐expression risk gene, and targeting UBE2C may offer therapeutic benefits and improve patient survival. Collectively, these findings underscore the potential of FAST treatment, which presents a variety of CTA, TSA, and TAA.

### 2.6 FAST Shows a Relatively Greater Potential in Pre‐Clinical Therapy Scenarios

**Figure 6:**
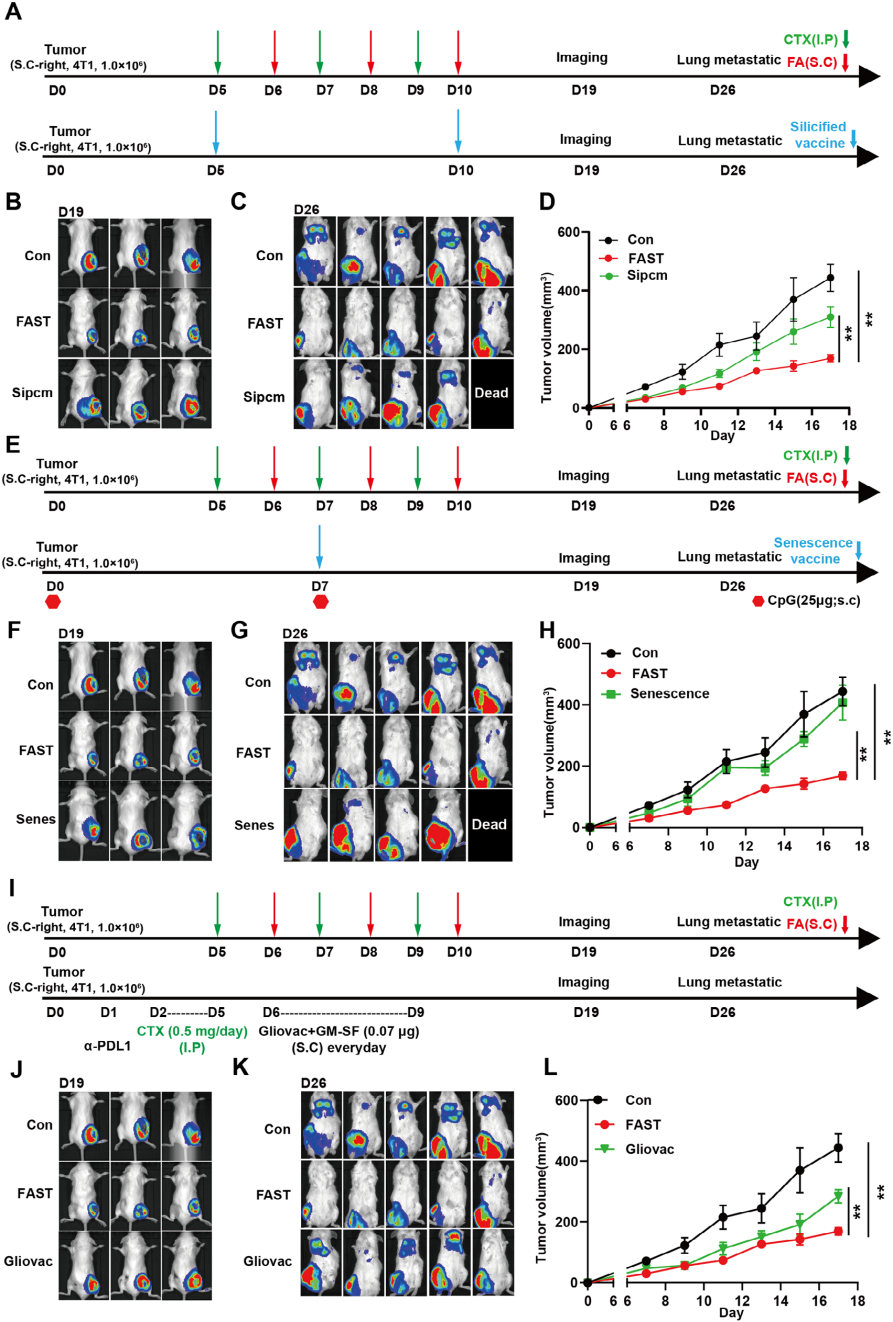
FAST demonstrates relatively superior potential within the realm of pre‐clinical therapy compared to three other TCVs (Silicified cancer vaccines (A), senescent cell vaccines (B), and Gliovac (C)). (A) The experimental procedure schematic of FAST and Silicified vaccine treatment. (B–D) Dynamics of tumor progression in control, FAST and Silicified vaccine treated group. (B) In vivo imaging of tumor volume in mice on day 19 after FAST and Silicified vaccine treatment. (C) Lung metastasis imaging in mice on day 26 after FAST and Silicified vaccine treatment. (D) Tumor volume statistics in mice after FAST and Silicified vaccine treatment. (E) Modeling protocol for FAST and Senescence vaccine treatment. (F–H) Dynamics of tumor progression in control, FAST and Senescence vaccine treated group. (F) In vivo imaging of tumor volume in mice on day 19 after FAST and Senescence vaccine treatment. (G) Lung metastasis imaging in mice on day 26 after FAST and Senescence vaccine treatment. (H) Tumor volume statistics in mice after FAST and Senescence vaccine treatment. (I) Modeling protocol for FAST and Gliovac vaccine treatment. (J–L) Dynamics of tumor progression in control, FAST and Gliovac vaccine treated group. (J) In vivo imaging of tumor volume in mice on day 19 after FAST and Gliovac treatment. (K) Lung metastasis imaging in mice on day 26 after FAST and Gliovac treatment. (L) Tumor volume statistics in mice after FAST and Gliovac treatment.

To evaluate the clinical potential of FAST therapy, we conducted a comparative analysis of its effectiveness against the latest clinical and pre‐clinical whole tumor cell vaccines. Significantly, our findings showed that FAST surpassed Silicified vaccines^[^ ^5^ ^]^ in controlling distal tumors (**Figure** **6**A–D). Moreover, on day 26, FAST exhibited a substantially greater ability to suppress lung metastasis compared to the Silicified vaccine (Figure 6C; Figure S13, Supporting Information).

When pitted against senescent‐cell vaccines,^[^ ^6^ ^]^ FAST demonstrated better control over distant tumors (Figure 6E–H). Interestingly, both therapies were equally effective in preventing lung metastasis (Figure 6G). In comparison with the Gliovac vaccine,^[^ ^40^ ^]^ which is currently in Phase II clinical trials, FAST showed a strikingly superior capacity to inhibit lung metastasis (Figure 6K) with similar efficacy in controlling distant tumors (Figure 6I–L).

In summary, compared to the most advanced pre‐clinical and Phase II clinical whole tumor cell vaccines, FAST provides enhanced control over both distant and metastatic tumors. This suggests its great potential for clinical application. Consequently, we initiated the clinical trial NCT06756295 and pursued ethical approval.

## 3 Conclusion and Discussion

Presently, we have developed the FAST strategy to rapidly prepare personalized cancer vaccines in under 7 h. This strategy is meticulously designed to enhance anti‐tumor immune responses (Figure 1). It entails ICD in tumor cells through ex vivo irradiation combined with cryoablation cycles (Figures 2 and 3). Moreover, it promotes a favorable anti‐tumor immune environment by remodeling the TIME and STIE (Figure 2). The FAST strategy not only preserves all tumor antigens, but also upregulates the intrinsic potential antigens of tumor cells (Figures 4 and 5). Significantly, multiple administrations of the FAST treatment induce immune memory in vivo and exhibit remarkable efficacy in inhibiting tumor metastasis (Figure 1). When compared with traditional and state‐of‐the‐art whole tumor cell vaccines, the FAST approach shows relatively greater potential in terms of pre‐clinical therapeutic efficacy (Figure 6).

Tumor cells are typically heterogeneous and constantly evolving, thereby evading vaccine attacks by altering antigen expression. Consequently, vaccines need to cover as many antigens as possible. Although tumor vaccines using well‐defined antigens have been explored for cancer immunotherapy, a significant challenge arises due to the limited number of shared antigens across different cancer types.^[^ ^3^ ^]^ This is primarily due to the inherent heterogeneity, complexity, diversity, and mutagenicity of antigens among patients, which hinders their widespread clinical application.^[^ ^41^, ^42^ ^]^ In contrast, whole tumor cell vaccines, which encompass all potential antigens, hold greater promise for inducing immune responses against cancer‐specific tumor antigens.^[^ ^3^ ^]^ The results from clinical trials NCT00045968, NCT03400917, and NCT02033616 suggest that ex vivo vaccines using broad‐spectrum antigens hold promise due to their potential to present the full range of tumor antigens, compared to predefined antigen vaccines. Additionally, these vaccines have demonstrated efficacy in inducing systemic tumor regressions.^[^ ^3^ ^]^ However, the major obstacle for their success remains the lack of whole‐cell immunogenicity. Currently, the immunogenicity of FAST is achieved by both amplifying the antigen signals upregulated during the FA construction process and remodeling the TIME and the STIE following multiple vaccinations.

On one hand, currently, multi‐omics analysis has identified 388 proteins that are upregulated in FAST vaccine antigens, among which 214 proteins are highly expressed in tumors. Notably, 20 of these proteins are upregulated at both the protein and transcriptional levels. After FAST treatment, the expression of these 20 proteins shows a downward trend in tumor tissues. These findings suggest that the high expression of these tumor antigens in FAST vaccines can effectively activate the immune system, enabling immune cells to target and eliminate tumor cells expressing these proteins, thereby inhibiting tumor growth.

In addition, In cancer immunotherapy, the accurate recognition of tumor antigens presented on MHC‐I molecules by cytotoxic CD8+ T cells is crucial for initiating effective antitumor immune responses.^[^ ^24^, ^25^ ^]^ Activation of CD8+ T cells requires APCs to activate naive precursor T cells, which then recognize tumor antigens bound to MHC‐I complexes via their TCRs. This process generates antigen‐specific signals that are essential for durable adaptive antitumor immunity.^[^ ^43^ ^]^ Presently proteomic analysis of tumor tissues before and after FAST treatment has revealed 680 differentially expressed proteins, of which 9 were downregulated and 12 were upregulated. TAP1, a key transporter involved in presenting tumor antigens via the MHC‐I complex, was markedly overexpressed in most cancers.^[^ ^44^ ^]^ Interestingly, TAP1 was among the 33 downregulated proteins following FAST treatment, with a marked reduction in tumor cells confirmed by ELISA assays. Moreover, MHC‐I antagonism was found to diminish the anti‐tumor effects of FAST, highlighting the critical role of MHC‐I in mediating these effects in FAST.

On the other hand, we have also demonstrated that FAST may reshape the TIME by enhancing the presence of effector T cells and inhibiting Treg cells in tumor tissues. Concurrently, the systemic microenvironment is reconfigured, FAST promotes the generation of CTLs through vaccine‐based immunotherapy, facilitates the release of anti‐tumor cytokines, and weakens immunosuppressive cells. Moreover, the results of key immune cell populations within both the TIME and the STIE indicate that CD8+ T cells play a pivotal role in controlling distant tumors, while both NK cells and CD8+ T cells contribute significantly to the control of disseminated metastasis. Interestingly, CD4+ T cells may exert a counteractive effect on metastasis. These results underscore the importance of immune system remodeling and the precise modulation of immune cell subsets as potential strategies for enhancing therapeutic outcomes and mitigating metastatic progression in the context of FAST.

Antigens of cancer cells tend to constantly change or evolve over time and with the evolution of the microenvironment. To address the potential immune escape resulting from these changes, the production of cancer vaccines should be expedited as soon as possible. Tumor antigens, especially neoantigens, are difficult to determine precisely, with low prediction efficiency, and the process is also time‐consuming, often taking several weeks or even several months. Unlike most advanced cancer vaccines, such as mRNA neoantigen vaccines or peptide neoantigen vaccines, which require precise antigen identification, FAST do not require precise pre‐identification of the key antigens of tumor cells. FAST bypass this complex antigen screening process and directly use the entire tumor cells to induce an immune response. Notably, FAST only takes 7 h to prepare personalized vaccines from sampling to injection, suggesting its potentially high clinical accessibility.

In summary, FAST may not only preserve all cancer cell antigens but does so to an even greater degree. In contrast to CAR‐T therapies or DC vaccines, it eschews the highly exacting ex vivo modification, culture, and selection processes of immune cells. Furthermore, unlike mRNA or peptide neoantigen vaccines, it obviates the need for sequencing, as well as the still suboptimal accuracy processes of neoantigen prediction, synthesis, and carrier delivery. Consequently, it is likely to offer clinical advantages that are urgently required in cancer therapy, such as personalization, rapid production, low cost, and high antigen coverage. We are eagerly looking forward to the clinical trial NCT06756295 associated with this research making the desired progress.

## 4 Experimental Section

### Animals

BALB/c WT and C57BL/6 WT were purchased from the Experimental Animal Center of Zhejiang Province and housed at the animal facility of Wenzhou Institute, University of Chinese Academy of Sciences. All experiments were reviewed and approved by the Institutional Animal Care and Use Committee of Wenzhou Institute, University of Chinese Academy of Sciences.

### Cell Lines

4T1‐Luc cells and CT26‐GFP^+^‐Luc cells were maintained at 37 °C with 5% CO_2_ in RPMI‐1640 supplemented with 10% FBS and 1% penicillin and streptomycin. B16‐F10‐GFP^+^‐LUC cells were maintained at 37 °C with 5% CO_2_ in DMEM supplemented with 10% FBS and 1% penicillin and streptomycin. CT26‐GFP^+^‐Luc cells and B16‐F10‐GFP^+^‐Luc cells were purchased from Shanghai Zhong Qiao Xin Zhou Biotechnology Co., Ltd. 4T1‐Luc cells were a gift from Wenzhou Medical University.

### Lymphocyte Extraction

Tumor tissue or fresh blood was collected, and lymphocytes were isolated and extracted using the Kit (Solarbio, P9000; Solarbio, P8620). Preparation of single‐cell suspension; Add an equal amount of separation solution to the single‐cell suspension; Carefully draw the single‐cell suspension and add it to the separation liquid surface, paying attention to maintaining a clear interface between the two liquid surfaces. Room temperature, 500–900 g, centrifuge for 20–30 min; Extract the second layer of circular milky white lymphocytes, wash milky white lymphocytes with washing solution, 250 g, centrifuge for 10 min.

### FAST Preparation

Tumor cells (1 × 10^7^) in vitro were washed with PBS, suspended in 1 mL physiological saline, and then irradiated with 12 Gy X‐rays at a dose rate of 6 Gy min^−1^. After irradiation for 1 or 6 h, tumor cells were repeatedly frozen and thawed using liquid nitrogen to eliminate tumorigenicity and promote antigen release. The tumor cell suspensions were then stored at −80 °C to completely lyse the tumor cells. Cyclophosphamide (CTX) as the immune adjuvant and irradiated tumor cell lysates were combined to form the FAST.

### Tumor Models and Treatment

Postsurgery tumor model: Six‐week‐old BALB/c were subcutaneously injected with 4T1‐Luc breast cancer cells in left flank. On Day 7, when the tumor volume reached ≈150 mm^3^, the mice were anesthetized with Inhaled isoflurane, and the tumor tissues were excised. On day 8, the mice were randomly divided into four groups, and they were injected with normal saline, Adjuvant, FA or FAST. For six consecutive days, CTX was intraperitoneal injected on days 1, 3, and 5, and FA was subcutaneously injected on days 2, 4, and 6. FA was immunized for three times. Mouse survival was monitored for 50 days.

In vivo therapeutic model: Six‐week‐old BALB/c and BC57BL/6 mice were subcutaneously injected with 4T1‐Luc cells or CT‐26‐Luc cells (1 × 10^6^ cells in 100 µL of normal saline per mouse) and B16F10 cancer cells (5 × 10^5^ cells in 100 µL of normal saline per mouse) in the right flank, respectively. Days 7, the mice were randomly divided into different groups. On days 6, 8, and 10, the mice were intradermally injected with normal saline or Irradiated tumor cell lysates (FA). The day before each cell lysate inoculation, mice were intraperitoneally inoculated with CTX at 50 mg kg^−1^ on days 5, 7, and 9. Tumor size was measured every 3 days using a digital caliper and computed according to the ellipsoidal calculation formula: V = 0.5 × (longest diameter) × (shortest diameter)^2^. Mouse survival was monitored for 50 days. The mice bearing tumors exceeding 2000 mm^3^ in size were euthanized.

Combined immunotherapy model: Six‐week‐old BALB/c mice were subcutaneously injected with 4T1‐luc cells in the right flank. Days 7, the mice were randomly divided into different groups. Immunity inhibitors (InVivoPlus anti‐mouse PD‐L1 (B7‐H1; BIOCELL, Catalog #BE0101) or InVivoPlus anti‐mouse CTLA‐4 (CD152; BIOCELL, Cat #BP0164) was intraperitoneal injected on days 5, 7, 9, and 11. FAST immunization was performed as in the therapeutic model. Tumor size was measured every 3 days using a digital caliper.

Antagonism of IFNγR and inhibition of the PI3Kδ pathway model: Six‐week‐old BALB/c mice were subcutaneously injected with 4T1‐luc cells in the right flank. On days 8, 10, 12, and 14, IFNγR antagonistic antibody (Cat:BE0029, BioXcell) (0.5 mg each) was intraperitoneally injected. Since the anti‐IFNγR antibody was a protein or monoclonal antibody with low absorption efficiency through the gastrointestinal tract, intraperitoneal injection was chosen. Inhibition of the PI3Kδ model: From day 8 to day 14, the PI3Kδ inhibitor Nemiralisib (Cat: S7937, Selleck) at a dose of 3 mg kg^−1^ was administered to mice by oral gavage daily.

### Bioluminescence Images

To obtain bioluminescence images, prior to imaging, D‐luciferin potassium salt (Aladdin, Shanghai, China) in sterile water was injected intraperitoneally according to the manufacturer's protocol. Bioluminescence images were acquired by IVIS spectrum computed tomography (Perkin Elmer), and the total luminescence flux in tumor tissues or lung tissues was quantified using Living Image 3.1 software.

### BMDC Harvest

Mouse bone marrow‐derived dendritic cells (BMDCs) were generated and modified as previously described.^[^ ^45^, ^46^ ^]^ Briefly, mouse bone marrow tissues were collected from BALB/c mice in a sterile environment, and erythrocytes were removed. The bone marrow cells were cultured in 30 mL complete RPMI 1640 medium supplemented with 20 ng mL^−1^ GM‐CSF. Two days later, 10 mL of fresh medium was added to the original medium. On day 5, the floating cells were gently removed, and the culture replenished with fresh medium containing 20 ng mL^−1^ GM‐CSF and 20 ng mL^−1^ IL‐4. On day 7, the non‐adherent and loosely adherent DCs in culture were harvested and used as the source of dendritic cells for various vaccines. DCs were routinely generated in this manner and found to be mainly immature DCs with 85% of cells expressing CD11c^+^ and displaying the typical morphological features of DCs.

### Dendritic Cell Activation

The procedure for performing tumor cell lysate activation of BMDCs was performed as previously described.^[^ ^45^, ^46^ ^]^ Briefly, BMDCs were incubated for 24 or 48 h with cell lysate from irradiated or not tumor cell. LPS at 10 µg mL^−1^ was added to the medium for co‐cultivation DCs for another 24 or 48 h as positive control. Then, cell mixture was stained for 30 min at 4 °C with antibodies, including PE‐conjugated anti‐mouse CD86^+^ and APC‐Cy7‐conjugated anti‐mouse CD80^+^. Two antibodies were obtained from Biolegend (San Diego, CA). The percentages of mature BMDC were detected by flow cytometry with CD86^+^/CD80^+^ cells.

### DAMPs Determination

DAMPs released or exposed by tumor cells after irradiation were detected. The secretion levels of HMGB1 (Solarbio, Cat:SEKM‐0145), ATP (Beyotime Biotechnology, S0027), HSP70 (CUSABIO, A23013068), and HSP90 (CUSABIO, Cat:A22013067) were analyzed using kit according to the manufacturer's instructions. CRT exposure was detected by immunofluorescence. In brief, cells on circular glass coverslips were fixed with 4% (v/v) paraformaldehyde in PBS (Santa Cruz Biotechnology) for 15 min at room temperature. Cells were washed with PBS, permeabilized with 0.5% (v/v) Triton X‐100 for 10 min and blocked for 30 min with goat serum (Sigma–Aldrich). Cells were incubated with CRT primary antibodies for 24 h at 4 °C followed by incubation with secondary antibodies for 2 h at room temperature. Finally, the cells were observed and imaged under an inverted fluorescence microscope (Zeiss, Germany).

### Flow Cytometric Analyses

For tumor‐infiltrating lymphocyte analysis of tumor tissues, tumor tissues were harvested on day 27. Cell suspensions were prepared by enzymatic hydrolysis of tumor tissue. Flow cytometric analyses of tumor‐infiltrating lymphocytes and single‐cell suspensions and peripheral blood mononuclear cell (PBMC) were performed using the following antibodies. The following antibodies were used: anti‐CD45‐APC‐CY7 (BD, Cat:557659); anti‐CD45‐AF700 (BD, Cat:560510); anti‐CD3‐FITC (BD, Cat:553061); anti‐FOXP3‐PE (BD, Cat:560408); anti‐CD62L‐PE (BD, Cat:553151); anti‐IFN‐γ‐PE (BD, Cat:554412); anti‐CD4‐Percp‐cy5.5 (BD, Cat:550954); anti‐Ki67‐APC (Thermo, Cat:17‐5698‐82); anti‐CD44‐APC (BD, Cat:559250); anti‐CD8e‐APC‐CY7 (BD, Cat:557654); anti‐CD11c‐BV421 (Biolegend, Cat:117329); anti‐MHC‐II‐BV650 (Biolegend, Cat:107641); anti‐MouseCD73‐BV786 (BD, Cat:752737); anti‐CD39‐BV421 (BD, Cat:567105); anti‐CD107a‐PE/Cy7 (Biolegend, Cat:121619); anti‐CD80‐APC (Biolegend, Cat:104713); anti‐CD86‐PE (Biolegend, Cat:159203); anti‐MHC‐I‐PE (BD, Cat:566776).

### Exhaustion Assay

Six‐week‐old BALB/c mice were subcutaneously injected with 4T1‐Luc cells in the right flank. The mice were randomly divided into each group. Mice were injected intraperitonially with 400 µg (in 100 µL PBS) of anti‐mouse CD4 (Cat:BE0003‐1, BioXcell), CD8β (Cat:BE0223, BioXcell), NK1.1 (Cat:BE0036, BioXcell) was administered on days 4, 7, and 10 prior to tumor cell vaccination. Isotype monoclonal antibody was using as control tumor cell vaccines (FAST) immunization was performed as in the therapeutic model. Subsequently, tumor size and lung tissue tumor metastasis were monitored to determine the role of different immune cells in anti‐tumor activity.

### ELISA

After irradiated cells with X‐rays, supernatant or cell lysate was collected and the HMGB1/ATP/HSP70/HSP90 concentration was measured with the ELISA kit (Solarbio, BeiJing, CN) according to the manufacturer's protocol. Measured concentrations were normalized based on the number of viable cells.

### Proteomic Analysis

Total protein extracted from the different treatment of tumor tissue or cells. Protein Quality Test was implemented by implement, then proteins were digested through trypsin. DDA spectrum library construction and DIA mode identification using UHPLC‐MS/MS for tumor samples were performed in Novogene Co., Ltd. (Beijing, China). Label free Methods and LC‐MS/MS Analysis were Used to analyze cell protein by Novogene Co., Ltd. (Beijing, China). The protein quantitation results were statistically analyzed by T‐test. The proteins whose quantitation significantly different between experimental and control groups, (*p* < 0.05 and |log2FC| >1 [fold change, FC]), were defined as differentially expressed proteins (DEP). Gene Ontology (GO) and InterPro (IPR) functional analysis were conducted using the interproscan program against the non‐redundant protein database (including Pfam, PRINTS, ProDom, SMART, ProSite, PANTHER), and the databases of COG (Clusters of Orthologous Groups) and KEGG (Kyoto Encyclopedia of Genes and Genomes) were used to analyze the protein family and pathway. DEPs were used for Volcanic map analysis, cluster heat map analysis, and enrichment analysis of GO, IPR, and KEGG. The differential membrane proteins of FAST‐treated tumor tissue and FA, identified and analyzed differential proteins related to FAST anti‐tumor immunity were analyzed.

### Statistical Analysis

Statistical analysis was performed using an unpaired, two‐tailed Students’ *t‐*test. Statistical analyses were conducted with GraphPad Prism 9.0 (San Diego, CA). Differences in tumor metastasis mouse survival rate were determined by a log‐rank (Mantel‐Cox) test of the Kaplan–Meier survival curves. An unpaired Students’ *t*‐test, one‐way ANOVA with Tukey's multiple‐comparison test, two‐way ANOVA with Tukey's multiple‐comparison test was used as indicated for comparisons between the groups. (*, *p <* 0.05; **, *p <* 0.01; ***, *p <* 0.001; n.s, no significance).

## Conflict of Interest

The authors declare no conflict of interest.

## Supporting information



Supporting Information

## Data Availability

The data that support the findings of this study are available from the corresponding author upon reasonable request.
