# Supplementary material for: Fragment Autoantigens Stimulated T‐Cell‐Immunotherapy (FAST) as a Fast Autologous Cancer Vaccine
Source: Adv Sci (Weinh). 2025 Mar 26;12(26):2502937. doi: 10.1002/advs.202502937 (PMC12245027; doi:10.1002/advs.202502937)
Supplement: Supplementary file 1 — Supporting Information [file ADVS-12-2502937-s001.docx]

Supporting Information

**Fragment Autoantigens Stimulated T-cell-immunotherapy (FAST) as a Fast Autologous Cancer Vaccine**

*Yuan Li, Huiqin Chen, Qiaofeng Shen, Yingshuang Liu, Pingping Li, Yuqi Ma, Yugang Wang, Shengkai Li, Xueqing Yan, Liyu Liu, Jianwei Shuai, Min Wu, Qi Ouyang, Feng-Ming (Spring) Kong, Gen Yang**

**Figure. S1**(A-D) Supplementary Figure. 1 for the 4T1 model. (E) Percentage of central memory T cells and effector memory T cells after three rounds of FAST immunization. (F) FAST treatment protocol for the B16-F10 model. (G-I) In vivo imaging of B16-F10 (CON vs FAST); tumor volume change curve; survival curve of B16-F10 model mice after FAST treatment. (J-L) In vivo imaging of CT26 model; tumor growth curve at day 35; survival curve of CT26 model mice after FAST treatment.

**Figure. S2** (A) Modeling protocol for FAST combined with ICB treatment. (B-C) In vivo imaging of FAST combined with α-PD-L1 treatment for distal tumors; tumor growth curve and lung metastasis risk. (D-E) In vivo imaging of FAST combined with α-CTLA treatment for distal tumors; tumor growth curve and lung metastasis risk. (F-G) Representative images of lung metastasis after FAST combined with α-PD-L1 or α-CTLA treatment.

**Figure. S3** (A) Percentage of KI67+ T cells in the tumor microenvironment after Con, FA, CTX, FAST treatment. (B) Percentage of IFNγ+ T cells in the tumor microenvironment after Con, FA, CTX, FAST treatment. (C-F), Immune microenvironment of the lungs after FAST treatment. (C) Percentage of lung CD3+ T cells in Con, CTX, and FA groups. (D) Percentage of lung CD8+ T cells in Con, CTX, and FA groups. (E) Percentage of lung CD4+ T cells in Con, CTX, and FA groups. (F) Percentage of lung CD73+ cells in Con, CTX, and FA groups. (G) Percentage of lung CD39+ cells in Con, CTX, and FA groups. (H) Typical flow cytometry plots of the lung microenvironment after FAST treatment (CD3+T; CD8+T; CD4+T; CD73+T;CD39+T).

**Figure. S4** (A) Heatmap of transcriptome expression profiles in tumor tissue after FAST treatment.

**Figure. S5** (A) KEGG enrichment analysis of 22 proteins selected from tumor tissue after FAST treatment. (B) GO enrichment analysis of 22 proteins selected from tumor tissue after FAST treatment.

**Figure. S6** Expression and influence analysis of FAST key genes in immunotherapy patients. (A**)** Expression of PI3Kδ in patients responding to ICB immunotherapy in clinical practice. (B) Expression of IFNGR1 in patients responding to ICB immunotherapy in clinical practice.

**Figure. S7** (A)Impact of Hazard Ratio and PI3Kδ expression levels on patient survival prognosis in immunotherapy-treated patients. (B) Impact of Hazard Ratio and IFNGR1 expression levels on patient survival prognosis in immunotherapy-treated patients.

**Figure. S8** (A-D)Secretion of ICD-related indicators (HMGB1, ATP, HSP70, HSP90) in tumor cells at different time points after radiation. (E) Representative immunofluorescence images of CRT (calreticulin) expression after irradiation. (F) Representative images and statistical analysis of induced DC (dendritic cell) maturation with LPS as a positive control.

**Figure. S9**(A) OS (overall survival) heatmap of 20 proteins across 12 different cancer types. (B) Impact of high expression of multiple genes from the 20 genes on OS prognosis in patients with four types of cancer. (C) DFS (disease-free survival) heatmap of 20 proteins across 12 different cancer types. (D) Impact of high expression of multiple genes from the 20 genes on DFS prognosis in patients with four types of cancer.

**Figure. S10** (A) The expression levels of six proteins in normal tissues were analyzed based on the GTEx database, and are ordered from low to high from left to right（The expression levels of testicular tissue-related proteins are indicated within the red box.）.

**Figure. S11** (A) The differential expression genes in the TCGA-BRCA dataset were identified using four different algorithms, and the results are presented in a Venn diagram. (B) The expression levels of UBE2C protein in normal tissues were analyzed based on the GTEx database, ordered from low to high from left to right. (C) Paired expression analysis of UBE2C in patient samples from the TCGA pan-cancer dataset.

**Figure. S12** (A) The expression levels of 13 proteins in normal tissues were analyzed based on the GTEx database and sorted from left to right, with expression levels ordered from low to high across the different tissues（The expression levels of testicular tissue-related proteins are indicated within the red box.）.

**Figure. S13** (A) Statistical Analysis of Lung Metastasis Risk on Day 26 in Tumor-Bearing Mice Treated with FAST and Three Latest Whole-Cell Vaccines.
